# Development and validation of ^68^Ga-PSMA-11 PET/CT-based radiomics model to detect primary prostate cancer

**DOI:** 10.1186/s13550-022-00936-5

**Published:** 2022-09-30

**Authors:** Shiming Zang, Shuyue Ai, Rui Yang, Pengjun Zhang, Wenyu Wu, Zhenyu Zhao, Yudan Ni, Qing Zhang, Hongbin Sun, Hongqian Guo, Ruipeng Jia, Feng Wang

**Affiliations:** 1 Department of Nuclear Medicine, Nanjing First Hospital, Nanjing Medical University, Nanjing, China; 2 Department of Urology, Affiliated Drum Tower Hospital, Medical School of Nanjing University, Nanjing, China; 3 Department of Urology, Nanjing First Hospital, Nanjing Medical University, Nanjing, China

**Keywords:** 68Ga-PSMA-11, PET/CT, Prostate cancer, Radiomics

## Abstract

**Background:**

This study aimed to develop a novel analytic approach based on a radiomics model derived from ^68^Ga-prostate-specific membrane antigen (PSMA)-11 PET/CT for predicting intraprostatic lesions in patients with prostate cancer (PCa).

**Methods:**

This retrospective study included consecutive patients with or without PCa who underwent surgery or biopsy after ^68^Ga-PSMA-11 PET/CT. A total of 944 radiomics features were extracted from the images. A radiomics model was constructed using the least absolute shrinkage and selection operator (LASSO) algorithm with tenfold cross-validation in the training set. PET/CT images for the test set were reviewed by experienced nuclear medicine radiologists. The sensitivity, specificity, positive predictive value, negative predictive value, and area under the receiver operating characteristic curve (AUC) were calculated for the model and radiologists’ results. The AUCs were compared.

**Results:**

The total of 125 patients (86 PCa, 39 benign prostate disease [BPD]) included 87 (61 PCa, 26 BPD) in the training set and 38 (61 PCa, 26 BPD) in the test set. Nine features were selected to construct the radiomics model. The model score differed between PCa and BPD in the training and test sets (both *P* < 0.001). In the test set, the radiomics model performed better than the radiologists’ assessment (AUC, 0.85 [95% confidence interval 0.73, 0.97] vs. 0.63 [0.47, 0.79]; *P* = 0.036) and showed higher sensitivity (model vs radiologists, 0.84 [0.63, 0.95] vs. 0.74 [0.53, 0.88]; *P* = 0.002).

**Conclusion:**

Radiomics analysis based on ^68^Ga-PSMA-11 PET may non-invasively predict intraprostatic lesions in patients with PCa.

**Supplementary Information:**

The online version contains supplementary material available at 10.1186/s13550-022-00936-5.

## Background

Prostate cancer (PCa) is one of the most common cancers and the second leading cause of cancer-related deaths among men worldwide [1]. Transrectal ultrasound-guided biopsy is currently a standard method for making a definitive diagnosis in patients with suspected PCa based on an elevated prostate-specific antigen level and/or an abnormal digital rectal examination [2]. However, traditional 10-core or 12-core systematic biopsy could fail to detect some cases of PCa and may incorrectly grade the tumor because of down-staging [2, 3]. In addition, prostate biopsy may be associated with notable side effects, including bleeding, pain, and infection. A non-invasive imaging approach for detecting PCa is thus an attractive prospect, to spare patients from unnecessary biopsies and overtreatment.

Prostate-specific membrane antigen (PSMA) is a highly specific prostatic epithelial cell transmembrane protein that is highly expressed in most primary PCa [4, 5]. ^68^Ga-labeled PSMA inhibitors have been explored and translated successfully for the clinical diagnosis of PCa in the last decade [6, 7]. ^68^Ga-PSMA-11 has been proposed for use in positron emission tomography/computed tomography (PET/CT) examinations among patients with primary PCa, and has demonstrated higher sensitivity and specificity than magnetic resonance imaging (MRI) for the detection of both intraprostatic tumor focal lesions and metastasis [8–10]. However, PSMA PET/CT imaging data are usually analyzed manually by nuclear medicine specialists, based on experience, which is challenging. Notably, significant numbers of intraprostatic lesions might be missed by visual PET-image interpretation due to their small size or configuration [11]. Quantitative measures of PSMA expression are therefore necessary to allow risk stratification of patients with primary PCa.

Radiomics is an attractive approach that converts medical images into mineable high-dimensional data via the high-throughput extraction of abundant imaging features [12, 13]. These features include a variety of gene expression types that provide a more comprehensive description of the tumor characteristics, thus enabling researchers to obtain an effective signature to inform objective clinical decisions [14–17]. However, the predictive value of ^68^Ga-PSMA-11 PET/CT radiomics in patients with PCa has not been widely investigated.

We therefore aimed to perform a comprehensive analysis and develop a radiomics model based on ^68^Ga-PSMA PET/CT, and evaluate its diagnostic performance for the non-invasive prediction of PCa.

## Methods

### Patients and study design

Eligible consecutive patients who underwent ^68^Ga-PSMA-11 PET/CT between February 2019 and May 2021 were retrospectively enrolled in this study. The inclusion criteria were: (1) pathologically proven PCa or biopsy-proven benign prostate disease (BPD) with a follow-up of at least 6 months, the biopsy was performed utilizing a 12-core extended scheme under the guidance of transrectal ultrasonography, without consideration of PET/CT; (2) ^68^Ga-PSMA-11 PET/CT examination performed within 1 month before surgery or biopsy; and (3) no anti-tumor treatment received before PET/CT examination. Patients with positive regional lymph node were included in case they had surgery, while those with distant metastasis were excluded for their unavailable surgery specimen. Patients were divided randomly into a training set and test set at a ratio of 7:3. Pathologically proven PCa from radical prostatectomy was used as reference standard and dichotomized for radiomics model classification and radiologist assessment.

### PET/CT acquisition and visual assessment

All patients underwent PET/CT using a dedicated PET/CT system (United Imaging, uMI780, China) at 60 ± 5 min after intravenous injection of 2–2.3 MBq/kg ^68^Ga-PSMA-11 synthesized as previously described [18]. A non-enhanced CT scan (120 kV, mA modulation, pitch 0.988, slice thickness 3.0 mm, increment 1.5 mm) was obtained followed by a whole-body PET scan (3 min/bed, field of view 60 cm) in 3D mode (matrix 256 × 256) from the vertex to the proximal legs. Datasets were fully corrected for random coincidences, scatter radiation, and attenuation. PET image reconstruction used the ordered-subsets expectation maximization method. Attenuation corrections of the PET images were performed using data from CT scans. PET/CT fusion was performed using a workstation (uWS-MI, United Imaging).

The training and test sets were reviewed independently by two nuclear medicine radiologists (F.W and S.Y.A, with 10 and 8 years of experience in prostate PET/CT, respectively). The radiologists were completely blinded to the clinical information and were encouraged to decide if an intraprostatic PCa lesion was present or not using a four-point scale: 1, definite BPD; 2, probable BPD; 3, probable PCa; and 4, definite PCa. Inter-reader agreement was evaluated using the Cohen's kappa coefficient. Agreement between the radiologists was reached by consensus.

### Image segmentation

**Fig. 1:**
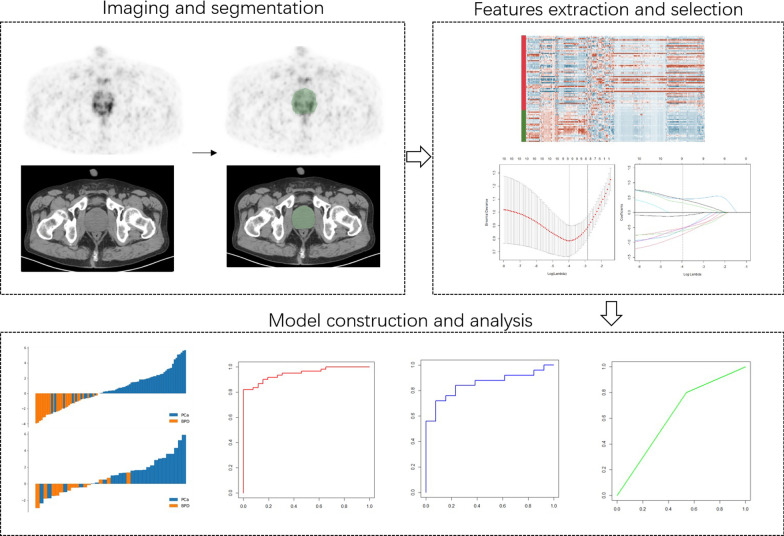
Radiomics modeling and analysis workflow

The radiomics workflow is shown in Fig. 1. The volumes of interest (VOIs) of the whole prostate gland were delineated manually and segmented slice by slice using 3D Slicer software (version: 4.1.1.0; www.slicer.org) by two nuclear medicine radiologists (Z.Y.Z. and S.M.Z., with 4 and 3 years of experience in prostate PET/CT, respectively), who were blinded to the clinical information, based on the PET images under consideration of the corresponding CT scan.

### Radiomics feature extraction

Radiomics features were extracted using the feature package of pyradiomics (github.com/Radiomics/pyradiomics) in Python, according to the guidelines of the Image Biomarker Standardization Initiative [19]. All PET data were subjected to image normalization and resampled to the same resolution (2 × 2 × 2 mm) before feature extraction (Additional file 1: Table S1). Standardized uptake value was discretized to a fixed bin width of 0.25. A total of 944 radiomics features were extracted, including 14 shape features, 18 first-order intensity statistics features, 75 texture features [Gray Level Co-occurrence Matrix (24), Gray Level Size Zone Matrix (16), Gray Level Run Length Matrix (16), Neighboring Gray Tone Difference Matrix (5), Gray Level Dependence Matrix (14)], and 837 wavelet and Laplacian of Gaussian features.

### Feature selection and radiomics model construction

**Fig. 2:**
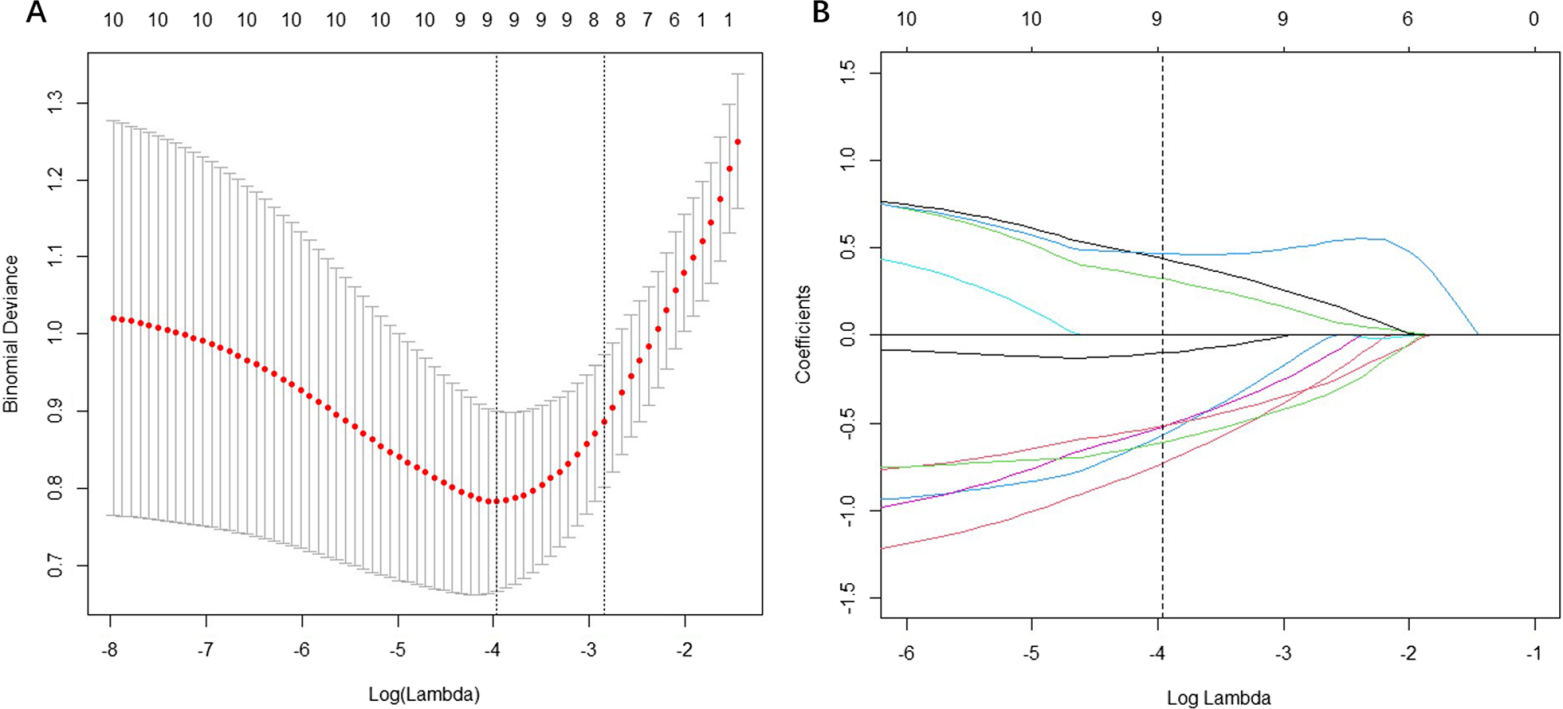
LASSO algorithm and tenfold cross-validation were used to extract the optimal subset of radiomics features. **a** Optimal tuning parameter (lambda) selection according to partial likelihood deviation of the model. **b** LASSO coefficient profiles of the 10 features. Using the tenfold cross-validation, a vertical line was drawn at the selected value and nine non-zero coefficients are shown

In this study, only radiomics features with good interobserver reproducibility (intraclass correlation coefficient [ICC] > 0.75) were included in subsequent analyses. Radiomics features were scaled using a z-score and reduced to 10 features by classic minimum redundancy maximum relevance using R2 difference. This algorithm ensures the selection of features that are highly relevant to the actual classes while reducing redundancy among the selected features. The minimum redundancy maximum relevance (mRMR) algorithm has been proven to be effective in both radiomics and genomics studies requiring the selection of a small subset of features from thousands of possible features [20, 21]. Finally, the least absolute shrinkage and selection operator (LASSO) algorithm was used to select significant distinguishable features with tenfold cross-validation (Fig. 2). To test the patient-based classification performance, a radiomics model score was then calculated for each patient in the training set and the test set using a formula constructed in the training set by a linear combination of selected features weighted by their respective coefficients.

### Statistical analysis

Differences in patients’ characteristics between the training and test sets were assessed. The ICC was calculated to evaluate the interobserver agreement among radiologists and interobserver reproducibility of the radiomics features, with an ICC > 0.75 indicating good reproducibility [22]. The sensitivity, specificity, positive predictive value (PPV), and negative predictive value (NPV) of the radiomics model and readers’ visual assessment were calculated. The diagnostic performances of the radiomics score and the readers’ evaluation for predicting PCa were evaluated using receiver operating characteristic (ROC) curve analysis. The optimal cutoff values of the radiomics score were determined by maximizing the Youden index in the training set. The fixed radiomics score cutoff values from the training set were then applied to the test set. The sensitivity, specificity, PPV, and NPV of the readers’ assessments were calculated by converting the four-point scale into a binary class. A score of 1 or 2 was regarded as BPD and a score of 3 or 4 was regarded as PCa. The AUC values were compared between the readers and radiomics model using the DeLong method.

Statistical analysis was conducted using SPSS 22.0 (IBM, Armonk, NY, USA) and R software (v. 4.1.3; http://www.Rproject.org). The LASSO regression was carried out using the “glmnet” package and ROC curves were analyzed using the “pROC” package. All statistical tests were two-sided, and a *P* value < 0.05 was considered statistically significant.

## Results

### Clinical characteristics

Among 237 patients, we excluded 91 because of anti-tumor treatment before PET/CT, 17 with distant metastasis and 4 because of history of malignancy. A total of 125 patients were finally included in the study and divided randomly into a training set (n = 87) and a test set (n = 38). All patients underwent biopsy or surgery and their pathological examination results were assessed. The baseline characteristics of the patients in the training and test sets are summarized in Table 1. The 87 patients in the training set included 61 (70.11%) with PCa and 26 (29.89%) with BPD, and the 38 patients in the test set included 25 (65.79%) with PCa and 13 (34.21%) with BPD. The distribution of ISUP grades was 1 (n = 8), 2 (n = 15), 3 (n = 21), 4 (n = 12), and 5 (n = 5) in the training set and 1 (n = 4), 2 (n = 4), 3 (n = 7), 4 (n = 6), and 5 (n = 4) in the test set. There were no significant differences in patient characteristics between the training and test sets.

**Table 1:** Clinical characteristics in the training and test sets

Characteristic	Training set (n = 87)	Test set (n = 38)	*P* value
Age (years)	71.00 ± 0.72	68.45 ± 1.24	0.076
PSA (ng/mL)	18.32 ± 2.25	18.72 ± 3.02	0.998
BPD	26 (29.89%)	13 (34.21%)	0.631
PCa	61 (70.11%)	25 (65.79%)	0.631
*ISUP grade*			
1	8 (13.11%)	4 (16.00%)	0.994
2	15 (24.59%)	4 (16.00%)	0.558
3	21 (34.43%)	7 (28.00%)	0.564
4	12 (19.67%)	6 (24.00%)	0.654
5	5 (8.20%)	4 (16.00%)	0.493

Age and PSA presented as mean ± standard deviation

PCa, Prostate cancer; BPD, benign prostate disease; PSA, prostate-specific antigen; ISUP, International Society for Urological Pathology

### Feature extraction and selection

A total of 944 radiomics features were extracted in the present study. Of these, 226 features with interobserver ICCs < 0.75 were eliminated. Ten features with the least redundancy and the greatest correlation with the target label were retained using the mMRM method. Following tenfold cross-validation of the LASSO algorithm on the training set, nine features with non-zero coefficients emerged as the best features to construct the radiomics signature (Additional file 1: Table S2), and the equation for calculating the radiomics model score was as follows:

Radiomics model score = 1.6371213 − original_shape_Sphericity × 0.1003826 – original_glszm_SmallAreaLowGrayLevelEmphasis × 0.7303062 + log-sigma-3–0-mm-3D_ngtdm_Coarseness × 0.3241121 − wavelet-HHH_glszm_LowGrayLevelZoneEmphasis × 0.5685874 − wavelet-HHH_glszm_SizeZoneNonUniformityNormalized × 0.5211243 + wavelet-LLL_firstorder_Skewness × 0.4401625 − wavelet-LLH_firstorder_Skewness × 0.5177129 − wavelet-LLL_glszm_GrayLevelNonUniformity × 0.6099202 + wavelet-LLL_glszm_SmallAreaEmphasis × 0.4692401.

### Radiomics model analysis

**Fig. 3:**
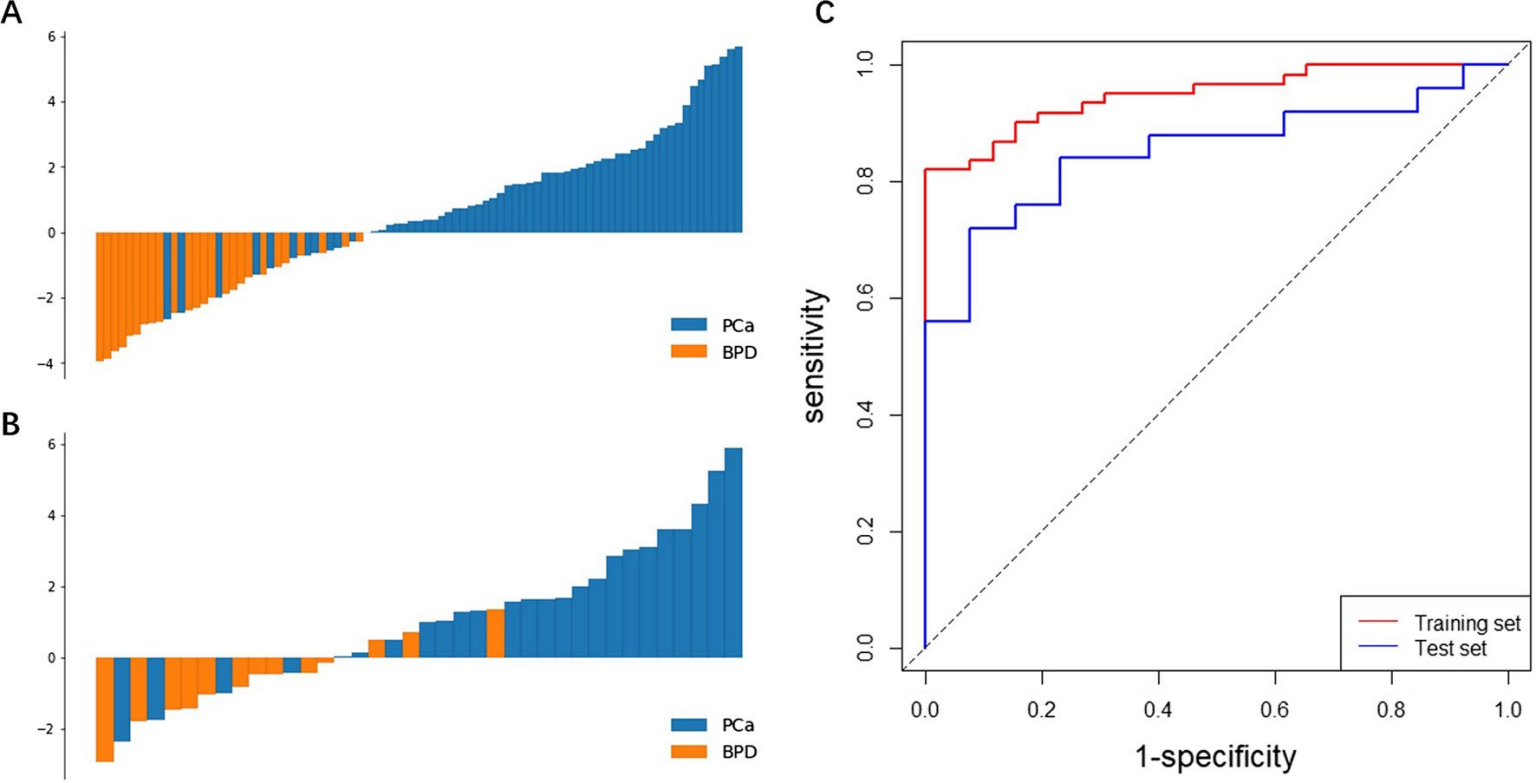
Diagnostic performance of the radiomics model. Model scores for patients in the **a** training and **b** test sets. **c** Receiver operating characteristic (ROC) curves of radiomics model in the training and test sets. PCa, prostate cancer; BPD, benign prostate disease

The performance of the radiomics features were evaluated by comparing the radiomics model scores based on the PET features. These differed significantly between the PCa and BPD groups in both the training and the test sets (both *P* < 0.001). Moreover, PCa had higher radiomics model score than BPD in both sets (Fig. 3).

The radiomics model had good predictive performance (Table 2), with sensitivity, specificity, PPV, and NPV values of 0.82 (95% confidence interval [CI]: 0.70, 0.90), 1.00 (95% CI: 0.84, 1.00), 1.00 (95% CI: 0.91, 1.00), and 0.70 (95% CI: 0.53, 0.84) in the training set, respectively. The equivalent values in the test set were 0.84 (95% CI: 0.63, 0.95), 0.77 (95% CI: 0.46, 0.94), 0.88 (95% CI: 0.67, 0.97), and 0.71 (95% CI: 0.42, 0.90), respectively. The AUC values for differentiating prostate histopathology were 0.95 (95% CI: 0.91, 0.99) and 0.85 (95% CI: 0.73, 0.97) in the training and test sets, respectively. Figure 3 shows the ROC curves of the radiomics model in the training and test sets.

**Table 2:** Diagnostic performances of the radiomics model and visual assessment by nuclear medicine radiologists

Cohort	Training set	Test set		
Category	Radiomics	Radiomics	Reader	*P* value
AUC (95% CI)	0.95 (0.91, 0.99)	0.85 (0.73, 0.97)	0.63 (0.47, 0.79)	0.036
Sensitivity (95% CI)	0.82 (0.70, 0.90)	0.84 (0.63, 0.95)	0.74 (0.53, 0.88)	0.002
Specificity (95% CI)	1.00 (0.84, 1.00)	0.77 (0.46, 0.94)	0.55 (0.25, 0.82)	0.508
PPV (95% CI)	1.00 (0.91, 1.00)	0.88 (0.67, 0.97)	0.80 (0.59, 0.92)	< 0.001
NPV (95% CI)	0.70 (0.53, 0.84)	0.71 (0.42, 0.90)	0.46 (0.20, 0.74)	0.754

AUC, Area under curve; PPV, positive predictive value; NPV, negative predictive value

**Fig. 4:**
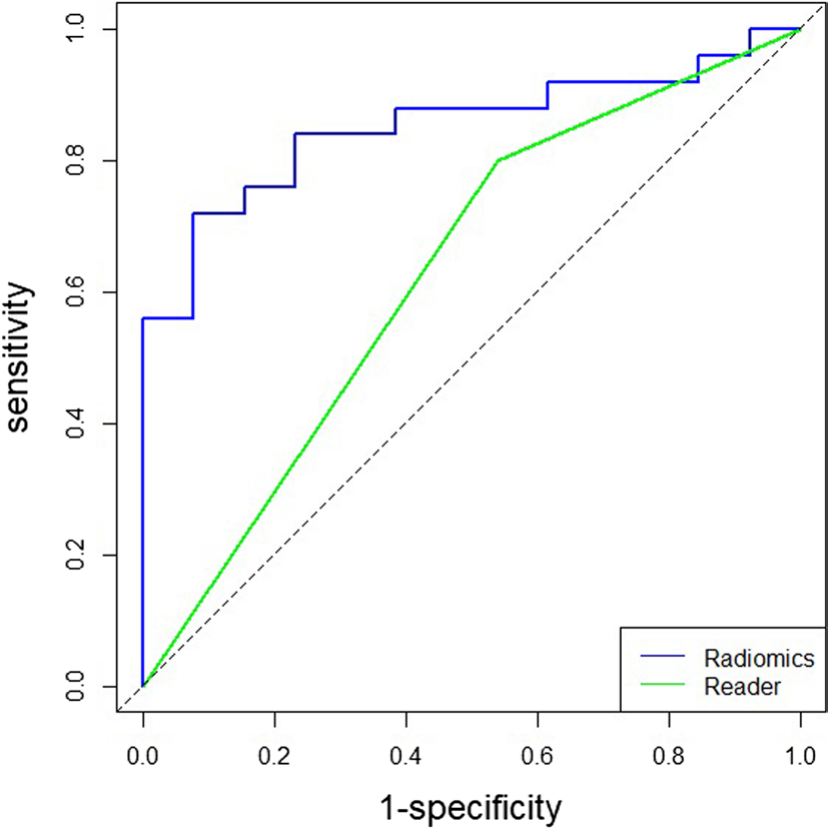
Receiver operating characteristic (ROC) curves of the radiomics model and visual assessment by nuclear medicine radiologists for discriminating PCa and BPD in the test set

The discriminatory efficiency of visual assessment by radiologists were also evaluated. Detailed information is provided in Table 2. The agreement between readers on was almost perfect (κ = 0.81 [95% CI: 0.59, 1.00]).The sensitivity, specificity, PPV, and NPV for the readers’ assessments were 0.74 (95% CI: 0.53, 0.88), 0.55 (95% CI: 0.25, 0.82), 0.80 (95% CI: 0.59, 0.92), and 0.46 (95% CI: 0.20, 0.74), respectively. Visual assessment demonstrated an AUC value of 0.63 (95% CI: 0.47, 0.79), which was significantly lower than the AUC value of the radiomics model (0.63 vs. 0.85, respectively; *P* = 0.036) (Fig. 4). The radiomics model also achieved greater sensitivity, specificity, PPV, and NPV than those of the readers (0.84 vs. 0.74 [*P* = 0.002], 0.77 vs. 0.55 [*P* = 0.508], 0.88 vs. 0.80 [*P* < 0.001], and 0.71 vs. 0.46 [*P* = 0.754], respectively).

## Discussion

In the present study, we developed a radiomics model based on ^68^Ga-PSMA-11 PET/CT for the non-invasive discrimination of patients with PCa from those with BPD. The model was successfully validated in independent test set (AUC, 0.85; sensitivity, 0.84; specificity, 77%; PPV, 0.88, NPV, 0.71) and outperformed visual assessments by nuclear medicine radiologists (AUC, 0.63; *P* = 0.036).

The non-invasive identification of patients with PCa is an important issue. Multi-parametric MRI (mp-MRI) has been an important diagnostic tool for detecting primary PCa for several years. Furthermore, the use of radiomics tools has improved radiologists’ assessments. Ginsburg et al. [23] evaluated features related to cancer detection in a transition zone and a peripheral zone in a cross-institutional setting and found that the radiomics features considered useful for cancer detection differed between the two zones. Cameron et al. [24] proposed a model consisting of an initial tumor candidate identification schema followed by the MAPS system (morphology, asymmetry, physiology, size) to score the candidate regions. The goal of the proposed model was to incorporate high-level features using candidate tumor regions through mp-MRI and region morphology to construct a high-dimensional feature space that could be mined for different purposes, such as cancer detection or prognosis prediction. However, these studies based on MRI do not reflect tumor heterogeneity as well as PSMA PET/CT, which targets a transmembrane glycoprotein substantially overexpressed in PCa cells [4, 5]. Zamboglou et al. [25] recently found that radiomics analysis of PSMA PET data was able to identify missing malignant lesions in the prostate gland. They enrolled patients with PCa and defined non-PCa tissue as the subtraction volume between the prostatic gland and PCa tumor, based on pathological tissue slices. However, further studies are needed to clarify the differences between radiomics features from the prostate tissue in the non-tumor area of prostate cancer patients and the prostate tissue in non-tumor patients. In the current study, we enrolled both PCa and non-PCa patients to comprehensively evaluate radiomics data from PSMA. Yi et al. [26] constructed a random forest model developed by ^68^Ga-PSMA-11 PET-based radiomics features proven to be useful for the accurate prediction of invisible intraprostatic lesions on ^68^Ga-PSMA-11 PET in patients with primary PCa (AUC, 0.903). Their study differed from the current study in that we evaluated both negative and non-negative PSMA-PET image cases. The present study showed a poorer model performance (AUC = 0.85), possibly due to differences in the inclusion criteria and region of interest between the two studies.

This study also compared the diagnostic performances of the radiomics model and qualitative evaluation by radiologists. Visual assessment of primary PCa based on experience remains challenging [27, 28]. Although PSMA is a transmembrane glycoprotein highly expressed on the cell surface of PCa cells, it is also expressed in benign pathologies such as BPD and prostatic intraepithelial neoplasia [29]. Benign intraprostatic processes can be associated with relatively high PSMA expression levels, with significant overlap between low-volume malignancies and benign disease. Moreover, visual PET interpretation might miss a significant number of intraprostatic lesions because of their small size or configuration [11], leading to potential false positives or false negatives [30, 31]. In this study, the radiomics model detected PCa based solely on a numeric feature set from high-dimensional medical imaging data, regardless of the clinical situation, which could explain its higher sensitivity compared with the readers’ assessments. On the other hand however, there was no significant difference in specificity, which might reflect the fact that the radiologists’ reports tended to maximize the specificity for an organ-preserving strategy, unlike the radiomics model determined by the Youden index. Further studies are thus needed to consider the specificity.

Whole-prostate gland segmentation strategy were employed in this study for two reasons. First, the characteristic large and low-resolution voxels for PET images limit the radiomics analysis in small prostate lesions, and VOIs with few voxels cannot provide much complementary information [32]. Second, it will be challenging to determine the value for negative or diffuse-pattern PET images. Recent studies [25, 26] showed that radiomics features derived from ^68^Ga-PSMA-11 PET images based on half-glandular segmentation were helpful for predicting invisible PCa lesions. Solari et al. [33] developed radiomics models based on delineating the whole prostate gland and showed good performances for predicting the postoperative Gleason score in PCa patients. In the present study, further comparison between the radiomics model and readers revealed that high-dimensional features offered better disease characterization for PCa.

This study had some limitations. First, its single-center design and relatively small sample size may compromise the model’s generalization ability and affect its sensitivity and specificity. It is therefore necessary to formulate a unified standard for multicenter studies and establish and test multicenter data using radiomics methods to improve the robustness of the model. Second, further studies using different PET/CT scanners are needed to validate the generalizability and robustness of the radiomics model. Third, we only analyzed PET-imaging data, and future research should aim to include multi-modal imaging data. Fourth, characterization of multifocality was not included in this study and is desirable in future research.

## Conclusions

In conclusion, we successfully developed and validated a radiomics model based on features extracted from ^68^Ga-PSMA-11 PET. This model provides a non-invasive and quantitative method for predicting intraprostatic lesions in patient with PCa.

## Supplementary Information

**Additional file 1: Table S1**. Configuration settings of radiomics in the study. **Table S2**. The nine features selected for the predictive model construction.

## Data Availability

All data presented in this study are available from the corresponding authors upon a reasonable request.

## Abbreviations

PSMA: Prostate-specific membrane antigen
mRMR: Minimum redundancy maximum relevance
LASSO: Least absolute shrinkage and selection operator
ICC: Intraclass correlation coefficient
ROC: Receiver operating characteristic

## References

[1] Siegel RL, Miller KD, Jemal A (2020). Cancer statistics, 2020. CA Cancer J Clin.

[2] Ahmed HU, El-Shater Bosaily A, Brown LC, Gabe R, Kaplan R, Parmar MK (2017). Diagnostic accuracy of multi-parametric MRI and TRUS biopsy in prostate cancer (PROMIS): a paired validating confirmatory study. Lancet.

[3] Mottet N, van den Bergh RCN, Briers E, van den Broeck T, Cumberbatch MG, De Santis M (2021). EAU-EANM-ESTRO-ESUR-SIOG guidelines on prostate cancer-2020 update. Part 1: screening, diagnosis, and local treatment with curative intent. Eur Urol.

[4] Serefoglu EC, Altinova S, Ugras NS, Akincioglu E, Asil E, Balbay MD (2013). How reliable is 12-core prostate biopsy procedure in the detection of prostate cancer?. Can Urol Assoc J.

[5] Paschalis A, Sheehan B, Riisnaes R, Rodrigues DN, Gurel B, Bertan C (2019). Prostate-specific membrane antigen heterogeneity and DNA repair defects in prostate cancer. Eur Urol.

[6] Sheikhbahaei S, Afshar-Oromieh A, Eiber M, Solnes LB, Javadi MS, Ross AE (2017). Pearls and pitfalls in clinical interpretation of prostate-specific membrane antigen (PSMA)-targeted PET imaging. Eur J Nucl Med Mol Imaging.

[7] Udovicich C, Perera M, Hofman MS, Siva S, Del Rio A, Murphy DG (2017). (68)Ga-prostate-specific membrane antigen-positron emission tomography/computed tomography in advanced prostate cancer: current state and future trends. Prostate Int.

[8] Perera M, Papa N, Roberts M, Williams M, Udovicich C, Vela I (2020). Gallium-68 prostate-specific membrane antigen positron emission tomography in advanced prostate cancer-updated diagnostic utility, sensitivity, specificity, and distribution of prostate-specific membrane antigen-avid lesions: a systematic review and meta-analysis. Eur Urol.

[9] Fendler WP, Calais J, Eiber M, Flavell RR, Mishoe A, Feng FY (2019). Assessment of 68Ga-PSMA-11 PET accuracy in localizing recurrent prostate cancer: a prospective single-arm clinical trial. JAMA Oncol.

[10] Donato P, Roberts MJ, Morton A, Kyle S, Coughlin G, Esler R (2019). Improved specificity with (68)Ga PSMA PET/CT to detect clinically significant lesions “invisible” on multiparametric MRI of the prostate: a single institution comparative analysis with radical prostatectomy histology. Eur J Nucl Med Mol Imaging.

[11] Donato P, Morton A, Yaxley J, Ranasinghe S, Teloken PE, Kyle S (2020). (68)Ga-PSMA PET/CT better characterises localised prostate cancer after MRI and transperineal prostate biopsy: Is (68)Ga-PSMA PET/CT guided biopsy the future?. Eur J Nucl Med Mol Imaging.

[12] Souvatzoglou M, Weirich G, Schwarzenboeck S, Maurer T, Schuster T, Bundschuh RA (2011). The sensitivity of [11C]choline PET/CT to localize prostate cancer depends on the tumor configuration. Clin Cancer Res.

[13] Gillies RJ, Kinahan PE, Hricak H (2016). Radiomics: images are more than pictures, they are data. Radiology.

[14] Lambin P, Rios-Velazquez E, Leijenaar R, Carvalho S, van Stiphout RG, Granton P (2012). Radiomics: extracting more information from medical images using advanced feature analysis. Eur J Cancer.

[15] Sun R, Limkin EJ, Vakalopoulou M, Dercle L, Champiat S, Han SR (2018). A radiomics approach to assess tumour-infiltrating CD8 cells and response to anti-PD-1 or anti-PD-L1 immunotherapy: an imaging biomarker, retrospective multicohort study. Lancet Oncol.

[16] Lambin P, Leijenaar RTH, Deist TM, Peerlings J, de Jong EEC, van Timmeren J (2017). Radiomics: the bridge between medical imaging and personalized medicine. Nat Rev Clin Oncol.

[17] Huang L, Lin W, Xie D, Yu Y, Cao H, Liao G (2022). Development and validation of a preoperative CT-based radiomic nomogram to predict pathology invasiveness in patients with a solitary pulmonary nodule: a machine learning approach, multicenter, diagnostic study. Eur Radiol.

[18] Lennartz S, O'Shea A, Parakh A, Persigehl T, Baessler B, Kambadakone A (2022). Robustness of dual-energy CT-derived radiomic features across three different scanner types. Eur Radiol.

[19] Zwanenburg A, Vallieres M, Abdalah MA, Aerts H, Andrearczyk V, Apte A (2020). The image biomarker standardization initiative: standardized quantitative radiomics for high-throughput image-based phenotyping. Radiology.

[20] Shi L, Shi W, Peng X, Zhan Y, Zhou L, Wang Y (2021). Development and validation a nomogram incorporating CT radiomics signatures and radiological features for differentiating invasive adenocarcinoma from adenocarcinoma in situ and minimally invasive adenocarcinoma presenting as ground-glass nodules measuring 5–10 mm in diameter. Front Oncol.

[21] Tang X, Liang J, Xiang B, Yuan C, Wang L, Zhu B (2022). Positron emission tomography/magnetic resonance imaging radiomics in predicting lung adenocarcinoma and squamous cell carcinoma. Front Oncol.

[22] Landis JR, Koch GG (1977). The measurement of observer agreement for categorical data. Biometrics.

[23] Ginsburg SB, Algohary A, Pahwa S, Gulani V, Ponsky L, Aronen HJ (2017). Radiomic features for prostate cancer detection on MRI differ between the transition and peripheral zones: preliminary findings from a multi-institutional study. J Magn Reson Imaging.

[24] Cameron A, Khalvati F, Haider MA, Wong A (2016). MAPS: a quantitative radiomics approach for prostate cancer detection. IEEE Trans Biomed Eng.

[25] Zamboglou C, Bettermann AS, Gratzke C, Mix M, Ruf J, Kiefer S (2021). Uncovering the invisible-prevalence, characteristics, and radiomics feature-based detection of visually undetectable intraprostatic tumor lesions in (68)GaPSMA-11 PET images of patients with primary prostate cancer. Eur J Nucl Med Mol Imaging.

[26] Yi Z, Hu S, Lin X, Zou Q, Zou M, Zhang Z (2022). Machine learning-based prediction of invisible intraprostatic prostate cancer lesions on (68) Ga-PSMA-11 PET/CT in patients with primary prostate cancer. Eur J Nucl Med Mol Imaging.

[27] Berger I, Annabattula C, Lewis J, Shetty DV, Kam J, Maclean F (2018). (68)Ga-PSMA PET/CT vs. mpMRI for locoregional prostate cancer staging: correlation with final histopathology. Prostate Cancer Prostatic Dis..

[28] Ferraro DA, Becker AS, Kranzbuhler B, Mebert I, Baltensperger A, Zeimpekis KG (2021). Diagnostic performance of (68)Ga-PSMA-11 PET/MRI-guided biopsy in patients with suspected prostate cancer: a prospective single-center study. Eur J Nucl Med Mol Imaging.

[29] Bostwick DG, Pacelli A, Blute M, Roche P, Murphy GP (1998). Prostate specific membrane antigen expression in prostatic intraepithelial neoplasia and adenocarcinoma: a study of 184 cases. Cancer.

[30] Ganeshalingam R, Hsiao E (2019). Compressed central zone uptake on PSMA PET/CT-A potential pitfall in interpretation. Clin Nucl Med.

[31] Pizzuto DA, Muller J, Muhlematter U, Rupp NJ, Topfer A, Mortezavi A (2018). The central zone has increased (68)Ga-PSMA-11 uptake: “Mickey Mouse ears” can be hot on (68)Ga-PSMA-11 PET. Eur J Nucl Med Mol Imaging.

[32] Hatt M, Majdoub M, Vallieres M, Tixier F, Le Rest CC, Groheux D (2015). 18F-FDG PET uptake characterization through texture analysis: investigating the complementary nature of heterogeneity and functional tumor volume in a multi-cancer site patient cohort. J Nucl Med.

[33] Solari EL, Gafita A, Schachoff S, Bogdanovic B, Villagran Asiares A, Amiel T (2022). The added value of PSMA PET/MR radiomics for prostate cancer staging. Eur J Nucl Med Mol Imaging.

