# Supplementary material for: Development and validation of 68Ga-PSMA-11 PET/CT-based radiomics model to detect primary prostate cancer
Source: EJNMMI Res. 2022 Sep 30;12:63. doi: 10.1186/s13550-022-00936-5 (PMC9522942; doi:10.1186/s13550-022-00936-5)
Supplement: Supplementary file 1 — Additional file 1: Table S1. Configuration settings of radiomics in the study. Table S2. The nine features selected for the predictive model construction. [file 13550_2022_936_MOESM1_ESM.docx]

**Additional File**

**Development and validation of ^68^Ga-PSMA-11 PET/CT-based radiomics model to detect primary prostate cancer**

**Shiming Zang^1#^, Shuyue Ai^1^**^#^**, Rui Yang^1^, Pengjun Zhang^1^, Wenyu Wu^1^, Zhenyu Zhao^1^, Yudan Ni^1^, Qing Zhang^2^, Hongbin Sun^3^, Hongqian Guo^2^*, Ruipeng Jia^3^* and Feng Wang^1^***

^1^ Department of Nuclear Medicine, Nanjing First Hospital, Nanjing Medical University, Nanjing, China

^2^ Department of Urology, Affiliated Drum Tower Hospital, Medical School of Nanjing University, Nanjing, China

^3^ Department of Urology, Nanjing First Hospital, Nanjing Medical University, Nanjing, China

^#^ These authors contributed equally to this work.

*** Corresponding author:**

Feng Wang

Email: fengwangcn68@163.com

Department of Nuclear Medicine, Nanjing First Hospital, Nanjing Medical University, Nanjing, China

Phone：86-25-52271491, Mobile Phone: 8618951670836

Ruipeng Jia

Department of Urology, Nanjing First Hospital, Nanjing Medical University, Nanjing, China

Email: ruipengj@163.com

Phone: 86-25-52271496, Mobile Phone: 8618951670931

Hongqian Guo

Department of Urology, Affiliated Drum Tower Hospital, Medical School of Nanjing University, Nanjing, China

Email: dr.ghq@nju.edu.cn

Mobile Phone: 8613605171690

**Table S1. Configuration settings of radiomics in the study**

| Normalize | Normalize scale | Resampled pixel spacing | Bin width | Interpolator | Precrop | Filter |
| --- | --- | --- | --- | --- | --- | --- |
| True | 1 | 2 × 2 × 2 | 0.25 | 3 | False | Laplacian of Gaussian/ Wavelet |

**Table S2. The nine features selected for the predictive model construction**

| Filter | Type | Feature | Formula * | Describe |
| --- | --- | --- | --- | --- |
| Original | shape | Sphericity | $\frac{\sqrt[3]{36\pi V^{2}}}{A}$ | Sphericity is a measure of the roundness of the shape of the tumor region relative to a sphere. It is a dimensionless measure, independent of scale and orientation. |
| Original | glszm | SmallAreaLowGrayLevelEmphasis | $\frac{\sum_{i=1}^{N_{g}} \sum_{j=1}^{N_{s}} \frac{P(i,j)}{i^{2}j^{2}}}{N_{z}}$ | SmallAreaLowGrayLevelEmphasis measures the proportion in the image of the joint distribution of smaller size zones with lower gray-level values. |
| Laplacian of Gaussian | ngtdm | Coarseness | $\frac{1}{\sum_{i=1}^{N_{g}} P_{i}s_{i}}$ | Coarseness is a measure of average difference between the center voxel and its neighbourhood and is an indication of the spatial rate of change. |
| Wavelet-HHH | glszm | LowGrayLevelZoneEmphasis | $\frac{\sum_{i=1}^{N_{g}} \sum_{j=1}^{N_{s}} \frac{P(i,j)}{i^{2}}}{N_{z}}$ | LowGrayLevelZoneEmphasis measures the distribution of lower gray-level size zones, with a higher value indicating a greater proportion of lower gray-level values and size zones in the image. |
| Wavelet-HHH | glszm | SizeZoneNonUniformityNormalized | $\frac{\sum_{j=1}^{N_{s}} {(\sum_{i=1}^{N_{g}} P(i,j))}^{2}}{N_{z}^{2}}$ | SizeZoneNonUniformityNormalized measures the variability of size zone volumes throughout the image, with a lower value indicating more homogeneity among zone size volumes in the image. |
| Wavelet-LLL | firstorder | Skewness | $\frac{\frac{1}{N_{p}}\sum_{i=1}^{N_{p}} {(X\left( i)-\bar{X} \right)}^{3}}{{(\sqrt{\frac{1}{N_{p}}\sum_{i=1}^{N_{p}} {(X\left( i \right)-\bar{X})}^{2}})}^{3}}$ | Skewness measures the asymmetry of the distribution of values about the Mean value. |
| Wavelet-LLH | firstorder | Skewness | $\frac{\frac{1}{N_{p}}\sum_{i=1}^{N_{p}} {(X\left( i)-\bar{X} \right)}^{3}}{{(\sqrt{\frac{1}{N_{p}}\sum_{i=1}^{N_{p}} {(X\left( i \right)-\bar{X})}^{2}})}^{3}}$ | Skewness measures the asymmetry of the distribution of values about the Mean value. |
| wavelet-LLL | glszm | GrayLevelNonUniformity | $\frac{\sum_{i=1}^{N_{g}} {(\sum_{j=1}^{N_{s}} P(i,j))}^{2}}{N_{z}}$ | GrayLevelNonUniformity measures the variability of gray-level intensity values in the image, with a lower value indicating more homogeneity in intensity values. |
| wavelet-LLL | glszm | SmallAreaEmphasis | $\frac{\sum_{i=1}^{N_{g}} \sum_{j=1}^{N_{s}} \frac{P(i,j)}{j^{2}}}{N_{z}}$ | SmallAreaEmphasis is a measure of the distribution of small size zones, with a greater value indicative of smaller size zones and more fine textures. |

glszm, Gray Level Size Zone Matrix; ngtdm, Neighbouring Gray Tone Difference Matrix.

** V* be the volume of the mesh; *A* be the surface area of the mesh; *N_g_* be the number of discreet gray levels; *N_s_* be the number of discreet zone sizes in the image; *N_p_* be the number of voxels in the image; *N_z_* be the number of zones; *P(i,j)* be the size zone matrix; *p_i_* be the gray level probability; *s_i_* be the sum of absolute differences for gray level; *X* be a set of *N_p_* voxels included in the region of interest.
